# Inter- and transgenerational epigenetic inheritance: evidence in asthma and COPD?

**DOI:** 10.1186/s13148-015-0085-1

**Published:** 2015-05-01

**Authors:** Susanne Krauss-Etschmann, Karolin F Meyer, Stefan Dehmel, Machteld N Hylkema

**Affiliations:** Comprehensive Pneumology Center, Helmholtz Center Munich and Children’s Hospital of Ludwig-Maximilians University, Max-Lebsche-Platz 31, 81377 Munich, Germany; Priority Area Asthma & Allergy, Leibniz Center for Medicine and Biosciences, Research Center Borstel and Christian Albrechts University Kiel, Airway Research Center North, Member of the German Center for Lung Research, Parkallee 1-40, Borstel, Germany; Department of Pathology and Medical Biology, University of Groningen, University Medical Center Groningen, Hanzeplein 1, Groningen, The Netherlands; University of Groningen, GRIAC Research Institute, University Medical Center Groningen, Hanzeplein 1, Groningen, The Netherlands

**Keywords:** Epigenetics, Transgenerational inheritance, Asthma, COPD

## Abstract

Evidence is now emerging that early life environment can have lifelong effects on metabolic, cardiovascular, and pulmonary function in offspring, a concept also known as fetal or developmental programming. In mammals, developmental programming is thought to occur mainly via epigenetic mechanisms, which include DNA methylation, histone modifications, and expression of non-coding RNAs. The effects of developmental programming can be induced by the intrauterine environment, leading to *inter*generational epigenetic effects from one generation to the next. *Trans*generational epigenetic inheritance may be considered when developmental programming is transmitted across generations that were not exposed to the initial environment which triggered the change. So far, inter- and transgenerational programming has been mainly described for cardiovascular and metabolic disease risk. In this review, we discuss available evidence that epigenetic inheritance also occurs in respiratory diseases, using asthma and chronic obstructive pulmonary disease (COPD) as examples. While multiple epidemiological as well as animal studies demonstrate effects of ‘toxic’ intrauterine exposure on various asthma-related phenotypes in the offspring, only few studies link epigenetic marks to the observed phenotypes. As epigenetic marks may distinguish individuals most at risk of later disease at early age, it will enable early intervention strategies to reduce such risks. To achieve this goal further, well designed experimental and human studies are needed.

## Review

### Introduction

Asthma and chronic obstructive pulmonary disease (COPD) are chronic lung diseases, both thought to result from a complex interplay of genetic factors and environmental exposures. These gene-environment interactions, in general, are now known to be mediated by epigenetic mechanisms such as histone modifications [1], DNA methylation [2] and hydroxyl methylation [3], chromatin remodeling [4], and expression of non-coding RNAs [5]. Epigenetic events are uniquely susceptible to endogenous and exogenous factors and most commonly take place during the prenatal period as the epigenome plays a vital role in embryonic development and tissue differentiation [6,7]. Epigenetic changes are different from genetic changes as they do not involve alterations of the DNA sequence and hence are, in principle, reversible [8]. As they were found to be inheritable, epigenetic events can be long lasting and passed on to the next generation. This is not limited to the first generation of the progeny but can involve the grandchildren and even further generations [9]. Mechanisms on epigenetic inheritance have been extensively reviewed recently [10-14] and will be discussed only briefly in this review.

#### Definition of inter- and transgenerational inheritance

In this review, we describe evidence from epidemiological and experimental studies for asthma and COPD which suggest that epigenetic inheritance does occur. However, epigenetic marks can only be retained and transmitted from one generation to the next when reprogramming of the germ line fails to remove epigenetic signatures that are required during development. The effects of developmental programming can be induced by the intrauterine environment (cigarette smoke, nutrition, and stress) which not only affect the fetus (F1) but also the germ line of the fetus (F2), leading to so-called intergenerational epigenetic effects. When developmental programming is transmitted across generations beyond F3, it is considered to be transgenerational and can not be explained by direct environmental exposure anymore. Only a few studies provide evidence for transgenerational epigenetic inheritance, which was mainly transmitted down the paternal line [9].

#### Epigenetics in asthma

Asthma is a common chronic inflammatory disorder of the airways, of which the prevalence has increased dramatically during the last two to three decades. Asthma is characterized by recurring episodes of airflow obstruction, intermittent chest symptoms such as wheezing, coughing, and shortness of breath, as well as bronchial hyperresponsiveness (BHR) [15,16]. In the developed world, approximately 50% of asthma patients suffer from the allergic phenotype of disease [17] in which type 2 helper T cell (Th2) activation is dominant, resulting in an increased level of Th2 cytokines, such as interleukin (IL)-4, IL-5, and IL-13, a decreased level of Th1 cytokines, such as interferon-gamma (IFN-γ), and an impaired function of regulatory T cells (Tregs).

Genetic sequence variations are associated with risk for asthma [18-23] but are *per se* unable to explain the escalating incidence of chronic inflammatory disorders during the past decades. Over time, it became evident that DNA variation can be associated with modified responses to environmental challenges [22]. However, genetic variants can also affect epigenetic signatures through differential DNA methylation of CPG sites [23,24]. Interestingly, a three-way interaction of genetic variations, DNA methylation and environmental exposure was first demonstrated by Salam *et al.* [25], who showed that exposure to particulate matter and methylation levels of the NOS2 promoter haplotypes jointly influenced levels of exhaled nitric oxide. Thus, epigenetic mechanisms in interaction with genetic variants might confer further flexibility towards environmental exposures.

Nonetheless, it remains an open question why environmental exposures interact with gene variations and thereby possibly bear potential to modify disease risks during critical developmental windows only. As outlined in the introduction, it has been hypothesized that environmental influences during vulnerable developmental periods may lead to lasting changes of the epigenome resulting in altered functionality of the lung and/or the immune system. So far, the majority of human studies have looked at associations of epigenetic modifications - for technical reasons mainly DNA methylation - with respiratory disease.

For example, genome-wide DNA methylation was analyzed in isolated peripheral monocytes from adult patients with eosinophilic, paucigranulocytic, or neutrophilic asthma *versus* healthy controls. While nine genes (TBX5, RBP1, NRG1, KCNQ4, PYY2, FAM19A4, SYNM, ME1, AK5) were hypermethylated and common to all asthma phenotypes, single *in silico* constructed networks were characteristic for the different asthma phenotypes [26].

In addition, using candidate gene approaches, a number of genes related to asthma and involved in oxidative stress, immunity, and lipid metabolism were investigated. In an analysis of 12 genes implicated in oxidative stress pathways, higher methylation of protocadherin-20 (PCDH-20) was observed in sputa from adult smokers with asthma compared to non-asthmatic subjects with a similar smoking history and without COPD [27]. Methylated paired box 5 protein transcription factor (PAX-5a), although not being associated with asthma risk, interacted synergistically with PCDH-20. In another study, adrenergic-receptor beta-2 (ADRB2) 5′-UTR methylation was analyzed in whole blood from 60 children with mild asthma and 122 children with severe asthma. Here, higher methylation was positively related to severity of asthma, in a dose-dependent manner [28]. In addition, children with severe asthma and exposure to higher levels of indoor NO_2_ correlated positively with ADBR2 methylation, indicating the latter may directly or indirectly modify the effect of NO_2_ on asthma severity. This observation was recently challenged by Gaffin *et al.* [29] who reported an inverse relation between average CpG methylation of ADBR2 with asthma severity in peripheral blood or saliva from 177 elementary school children with physician-diagnosed asthma, enrolled in the School Inner-City Asthma Study. The participants of both studies were of comparable age and similar diagnostic criteria for asthma were applied; however, as also emphasized by the authors, different regions of the ADRB2 gene were analyzed. This highlights the necessity to ensure that altered methylation affects gene expression and function and is not an epiphenomenon.

In this line, differential methylation of FOXP3 and IFNγ promoter regions was demonstrated in isolated peripheral regulatory and effector T cells from 21 monozygotic twin pairs discordant for asthma (age range 9 to 76 years). Higher methylation of both genes was associated with reduced mRNA and protein levels and was further associated with reduced suppressor function and T cell proliferation. Interestingly, FOXP3 levels were lowest in asthmatic twins who were additionally exposed to passive smoking. Moreover, increased methylation of FOXP3 was confirmed in purified bronchoalveolar lavage fluid (BALF) Tregs obtained from a subset of twins [30]. This indicates that relevant epigenetic changes of immune cells may also be seen in the periphery which would facilitate investigations in humans. On the other hand, Stefanowiscz *et al*. emphasized the importance of addressing epigenetic changes in relevant target tissues [31] while DNA methylation of STAT5A and CRIP1 in airway epithelial cells distinguished asthmatic children from non-asthmatic atopics and healthy controls, these differences were not observed in PBMC. Similarly, cell-specific DNA methylation at the A disintegrin and metalloprotease 33 (ADAM33) gene promoter, which has been implicated in severe asthma, differed considerably between epithelial cells and fibroblasts and resulted in altered gene regulation [32].

In peripheral B cells, the promoter region of prostaglandin D2 (PGD2) - an arachidonic acid-derived metabolite supporting Th2 cell differentiation and eosinophilia - was found to be hypomethylated from children with physician-diagnosed asthma compared to healthy controls [24]. Of note, the authors showed that hypomethylation was a) related to DNA variants and b) confirmed that this resulted in higher PGD2 expression levels supporting the functional relevance of these epigenetic changes.

Studies on epigenetics in asthma may have been hampered, as over the years, different clinical subgroups have been described. Hierarchical cluster analysis has demonstrated that there are at least five phenotypes which segregate according to age of onset, atopy, pulmonary function, requirement for medications, and a number of other factors [33]. However, in most published studies, rigorous phenotyping of patients is lacking.

#### Epigenetics in COPD

COPD is a life-threatening lung disease, mainly caused by cigarette smoking although other inhaled noxious particles and gases may contribute [34]. This leads to chronic airway inflammation, airway remodeling, and emphysema of the lung parenchyma. These lung pathologies lead to obstruction of lung airflow that interferes with normal breathing and is not fully reversible upon treatment [35]. Also for COPD, the evidence of epigenetic changes is emerging. Epigenetic modifications in bronchial epithelium and sputum have been linked to health status in patients with COPD [36,37] and cigarette smoking [38,39]. In addition, epigenetic regulation was found to be critically important in chronic remodeling [40], as well as in small airway pathology. In small airway epithelial cells (SAE) of nine ex-smoking patients with COPD, hundreds of genes were found predominately hypermethylated relative to SAE of ex-smoking individuals without COPD, which was associated with lower lung function [41]. Furthermore, as reviewed in [42], the expression of the different epigenetic patterns in various muscles of patients with COPD was found to explain skeletal muscle dysfunction, a possible systemic manifestation of this lung disease, especially in advanced stages of COPD.

Additionally, an epigenome-wide analysis in peripheral blood cells demonstrated a large number of differentially methylated genes, including the aryl hydrocarbon receptor repressor (AHRR) [43], which was confirmed together with F2RL3 later in an independent study [44]. Of note, differential methylation of the AHRR was also found in cord blood from children after prenatal smoke exposure [45] (see below) and was shown to persist until early infancy [46], demonstrating that there is at least in some instances a prolonged epigenetic memory of environmental insults.

#### Temporal changes of DNA methylation

Although the examples above illustrate that epigenetic changes occur in asthma and COPD, they bear the risk of reverse causation as epigenetic modifications are inducible and may represent a response to the pathology rather than being its root. Therefore, temporal changes of epigenetic marks as well as the timing of exposure and outcome need to be investigated during the life course. Temporal persistence of epigenetic DNA modifications were observed in adults after extended periods of smoking cessation in adults, which may explain the prolonged health risks after cigarette smoking. Thus, differential methylation of F2RL3 and GPR15 was shown to be significantly associated not only with current smoking but also with time since quitting smoking, in a dose-response relationship [47]. Similarly, Tsaprouni *et al.* reported reduced peripheral blood DNA-methylation which was only partially reversible after smoking cessation [48]. In addition, pet keeping and tobacco smoke exposure was shown to limit increase in CD14 methylation from 2 to 10 years of age in 157 children from the prospective Environment and Childhood Asthma birth cohort, partly explaining the diverging CD14 allele associations with allergic diseases detected in different environments [49].

With regard to asthma, genome-wide DNA methylation of CpG sites was recently assessed in peripheral blood leukocytes from 245 female participants of the Isle of Wight cohort at the age of 18 years [50]. In a subset of 16 and 18 women with and without asthma, DNA methylation was assessed in samples collected at 10 years of age. Focussing on genes of the Th2 pathway (IL4, IL4R, IL13, GATA3, STAT6), the authors demonstrated that the odds of asthma tended to decrease at the age of 10 years with increasing GATA3 methylation. This effect disappeared by age 18. Depending on the IL-4R genotype, methylation of two CpG sites was associated with higher risk of asthma in 18 year olds. These CpGs had no effect at the age of 10 years. Increasing methylation of one of the CpGs over time was related to a reduced risk of developing asthma in the first 10 years of life and increased the probability for 10-year-old asthmatics to have lost the disease by the age of 18. Thus, the study shows not only an interaction between IL-4R gene variants and DNA methylation in relation to asthma but also an effect of temporal change of DNA methylation on asthma transition between ages 10 and 18.

#### Prenatal exposures and epigenetic changes related to asthma or COPD risk

Numerous prenatal exposures such as maternal asthma or atopy, maternal nutrition or obesity during pregnancy, maternal gestational stress, and pollutants have been brought into context with respiratory disease. Among these, *maternal smoking* during pregnancy is one of the most important risk factors for impaired lung function development and asthma risk [51-53]. Since childhood asthma was shown to increase the risk for adult airflow obstruction by 20-fold [54], prenatal smoke exposure is also a potential risk factor for COPD.

Maternal smoking has been linked with genome-wide higher methylation of peripheral blood. In 92 adult women from a birth cohort dating back to 1959 (New York participants of the ‘National Collaborative Perinatal Project’) methylation of repetitive elements (LINE1-M1, Sat2-M1, Alu-M2), which are markers of global methylation in the identical blood samples, showed an inverse association between prenatal smoke exposure and Sat2 methylation [55]. In addition, an inverse dose-response relationship was observed between cord blood cotinine levels and global cord blood DNA hypomethylation in 30 newborns [56].

Breton *et al*. observed lower DNA methylation levels of the short interspersed nucleotide element AluYb8 in buccal cells from 348 prenatally exposed kindergarten and elementary school children [57]. Hypomethylation of LINE-1 was only seen in prenatally exposed children who were glutathione *S*-transferase (GST) M1 null, while methylation was higher in those with GSTM1. Thus, variants in detoxification genes may modulate the effects of prenatal exposure via differential epigenetic marks.

In candidate gene approaches, significant, albeit small differences in methylation of Neuropeptide S Receptor 1 (NPSR1) were observed in whole blood samples from adults with severe asthma and children with physician-diagnosed allergic asthma of a Swedish Birth cohort (BAMSE). In children, the NPSR1 methylation status was influenced by prenatal smoke exposure [58]. Prenatal smoke exposure throughout pregnancy was further associated with higher DNA methylation of the paternally expressed insulin-like growth factor 2 (IGF2) in cord blood when compared to samples from infants born to mothers who quit smoking in early pregnancy. There was a clear gender difference as methylation levels differed most significantly in male offspring [59]*.*

Within the Isle of Wight birth cohort, Patil *et al.* analyzed the interaction of six CpG sites in the IL-13 promoter with two functional gene variants of IL-13 in 245 female participants at the age of 18 years. The authors demonstrated a) an interaction of one functional IL-13 gene variant, rs20541, and maternal smoking during pregnancy with DNA methylation at one CpG site and b) that the interaction of this CpG site with another functional SNP affected airflow limitation and airway reactivity [2]. The authors propose a two-stage model where exposures first interact with so called methylation quantitative trait loci, that is, gene variants affecting susceptibility to DNA methylation, thereby modifying gene regulation. The response to subsequent environmental challenges potentially interacting with other gene variants within the same gene in a second stage would then be affected by the presence or absence of the epigenetic modification established during the first stage.

In an epigenome-wide association study (EWAS), lower cord blood DNA methylation of cytochrome P450 aryl-hydrocarbon-hydroxylase *(*CYP1A1) gene and hypermethylation of most investigated CpG sites of the aryl hydrocarbon receptor repressor gene (AHRR) were demonstrated in 1,062 prenatally smoke-exposed children of the Norwegian Mother and Child Cohort Study (MoBa) [45]. Both molecules play an important role in metabolizing xenobiotics and were also modified in adult smokers [60]. Thus, prenatal tobacco smoke exposure may predispose to altered responses towards xenobiotics in later life via lasting epigenetic modifications which could affect the risk for pulmonary disease. In contrast to the findings in cord blood, the identical CYP1A1 CpG sites were hypomethylated in placentae of smoking women with higher CYP1A1 mRNA expression [61]. Further changes of global DNA methylation in placental tissue from smoking mothers have been reported [62,63].

Another recent large EWAS identified and partly newly confirmed 185 CpG sites with altered methylation among FRMD4A, ATP9A, GALNT2, and MEG3, in whole blood of infants of smokers within 889 newborns from the Norway Facial Clefts Study.

These genes are implicated in processes related to nicotine dependence, smoking cessation, and placental and embryonic development [64]. In addition to blood sample analyses, recently, an EWAS was performed in 85 fetal *lung* and the corresponding placental tissue samples of which 41 were smoke exposed, using the Illumina HumanMethylation450 BeadChip array. Analyses of DNA methylation was conducted to evaluate the variation associated with nicotine exposure. The most significant differentially methylated CpG sites in the fetal lung analysis mapped to the PKP3, ANKRD33B, CNTD2, and DPP10 genes. In the placental methylome, however, the most significant CpG sites mapped to the GTF2H2C and GTF2H2D genes and 101 unique CpG sites were concordant between lung and placental tissue analyses. Gene Set Enrichment Analysis demonstrated enrichment of specific disorders, such as asthma and immune disorders, suggesting a role for DNA methylation variation in the fetal origins of chronic diseases [65].

Besides maternal smoking, prenatal *exposure to airborne pollutants* has been suggested as risk factor for asthma. High prenatal exposure to polycyclic aromatic hydrocarbons (PAH) has been reported to be associated with higher methylation of an enzyme involved in fatty acid metabolism termed acyl-CoA synthetase long-chain family member 3 (ACSL3) in cord blood DNA and matched fetal placental tissues [66]. The relation of ACSL3 function with asthma is not known. In a subsequent study, the authors reported hypermethylation of the IFNγ promoter in cord blood DNA in association with maternal PAH exposure [67]. Higher levels of prenatal dichlorodiphenyldichloroethylene, a metabolite from the pesticide DDT, were associated with DNA hypomethylation at age 4 years of a CpG site in the arachidonate 12-lipoxygenase (ALOX12) gene and associated with persistent wheezing in 6-year-old children from two independent Spanish cohorts. ALOX12 DNA methylation was further associated with genetic polymorphisms [68].

Intrauterine exposure to a *farming environment* has further been associated with decreased risk for asthma and allergies. The CD14 promoter region was differently methylated in placentae from women living on a farm compared to non-farming women [69].

In a birth cohort, cord blood Treg cell counts were increased with maternal farming exposures during pregnancy and associated with higher FOXP3 expression [70]. FOXP3 hypomethylation was increased with maternal consumption of farm milk. More recently, the Protection against allergy: study in rural environments (PASTURE) study was used to investigate methylation patterns of ten asthma candidate genes in cord blood and at age 4.5 years. ORMDL1 and STAT6 were hypomethylated in cord blood DNA from farmers’ offspring, while regions in RAD50 and IL-13 were hypermethylated [71]. An association with asthma was only seen in non-farming offspring for hypermethylated cord blood ORMDL3 and STAT6. Irrespective of the exposure or disease status, methylation of several asthma and allergy-related genes changed over time (IL-4, IL-13, ORMDL3, RAD50), indicating their involvement in developmental processes, while Treg-related genes (FOXP3, RUNX3) remained unchanged.

#### Exposures beyond the mother: what about fathers and ancestors?

Studies based on historical records of a small population in North Sweden (Överkalix) reported that the mortality rate of men is linked to food supply of the father’s father in mid-childhood, while the mortality rate of women was exclusively related to food supply of their father’s mother [72]. Data from the Avon Longitudinal Study of Parents and Children (ALSPAC) indicate an association of grandmaternal smoking with increased birth weight, birth length, and BMI in grandsons of non-smoking mothers but not in granddaughters. The same group of authors reported an association of paternal prepubertal smoking with greater BMI of their sons [73].

To date, there is very limited evidence for an exclusive establishment of risk for respiratory disease via the mother. Li *et al.* reported in 2005 that a grandchild’s asthma risk is increased if the grandmother smoked cigarettes during her pregnancy, even if the mother did not smoke [74]. So far, this issue has been investigated again in the ALSPAC where such an association was seen for the paternal, but not the maternal, grandmother [75]. Interestingly, this relation was stronger for granddaughter than for grandson’s asthma risk indicating again gender-specific effects. Epigenetic alterations were not investigated in these studies. Also in the Norwegian Mother and Child Cohort Study, grandmother’s smoking when pregnant with the mother was not associated with cord blood DNA methylation in the grandchild at loci associated with maternal smoking during pregnancy [76]. This however, does not exclude the possibility that the grandmother’s smoking is associated with DNA methylation in the grandchild in other areas of the genome.

#### Experimental intergenerational epigenetics

Animal models facilitate to investigate epigenetic inheritance across generations. So far, a number of prenatal exposure scenarios including maternal exposure to allergens [77], tobacco [78], nicotine [79,80], pollutants [81], bacteria or bacterial compounds [82], fungi [83], and maternal stress [84] have been investigated in intergenerational animal models for asthma risk (Table 1). Several studies report reduced lung function [78,84-87] and/or altered lung structure [78,87-89]. In addition, expression of genes with known or so far unknown relationship to asthma was investigated [90,91]. However, there is currently a paucity of studies aiming to investigate underlying epigenetic mechanisms [80,92]. Although several models included exposures during the preconceptional period [77,93-97], very few addressed effects on asthma risk during this period only [93,98].

**Table 1:** **Overview of experimental** ***in utero*** **exposure models**

**Exposure type**	**Preconceptional exposure**	**Prenatal exposure**	**Postnatal challenge**
Sidestream tobacco smoking (SS; ‘passive smoking’)	SS [87,99]	SS [85,87,89,91,99,100,126-133]	SS [85,87,89,91,99,100,126-133], OVA [91,130]
Mainstream tobacco smoking (MS; ‘active smoking’)	MS [78,86,90,102,103,134,135]	MS [78,86,90,101-103,134,135]	SS [86,134], *A. fumigates* [86], HDM [78], MS [103,135]
Active and passive tobacco smoking		MS + SS *vs*. SS [136]	
Surrogate for tobacco smoking	Nicotine [137], nicotine (F1*) [9,80]	Nicotine [79,138-142], nicotine (F1) [9,80]	Nicotine [79], nicotine (F1*) [9,80]
Pollutants	BPA [143], TDI or DNCB [144]	BPA [143,145,146], DEP [147-149] DEP, TiO_2_, or carbon black [150], ROFA [151], ozone [152], UPM [153], FA [154]	OVA [143,144,147,150,152-154], low- or high-fat diet [148], ozone [149]
Allergens	OVA [77,93,96,97,105,155-157], peanut [92]	OVA [77,96,155,158-160], OVA + Al(OH)_3_ OVA + PT, or OVA + CpG [161]	OVA [77,93,96,105,156-160], OVA or Casein [155], OVA or BLG [97], OVA + Al(OH)_3_ [161], peanut [92]
Bacteria and bacterial compounds	LPS [82]	LPS [82,162,163], LPS + iNOS inhibitor [164], *A. lwoffii* F78 [165,166], *L. rhamnosus* [167]	OVA [82,163-167], LPS, or OVA [162]
Fungi	*A. fumigates* [83,108], *M. anisopliae* [168]	*A. fumigates* [83], DEP + *A. fumigates* [169], *M. anisopliae* [168]	*A. fumigates* [83,108], *M. anisopliae* [168]
Nutrition, stress, and other factors	Methyl donor supplement [109], vitamin D3-deficient diet [170]	Noise [169], methyl donor supplement [109], vitamin A-deficient diet [171], vitamin D3-deficient diet [170], vitamin D-deficient diet [172], FBZ [173]	OVA [109,169], vitamin D3-deficient diet [170], vitamin D-deficient diet FBZ [172] and OVA [173]

Studies investigating effects up to F2: [80,83,109]. Studies investigating effects up to F3: [9,145]. Studies using rhesus monkeys: [126] (all other studies mentioned use rodents). Not all studies relate to a lung phenotype. *Studies with F1 animals exposed via mothers milk and used for further breeding. OVA, ovalbumin; HDM, house dust mite extract; BPA, bisphenol A; TDI, toluol-2,4-diisocyanat; DNCB, dinitrochlorobenzene; DEP, diesel exhaust particles; ROFA, residual oil fly ash; UPM, urban particulate matter; FA, formaldehyde; PT, pertussis toxin; BLG, beta-lactoglobulin; LPS, lipopolysaccharide; FBZ, fenbendazol.

Up to date, several intergenerational animal models exist which address fetal exposure to maternal passive smoking [85]. A study by Rouse *et al.* reported that *in utero* exposure to environmental tobacco smoke (ETS) did not alter the respiratory structure or function in offspring at the age of 10 weeks [91]. However, after ovalbumin (OVA) sensitization and challenge at the age of 10 weeks, lung function was impaired in both male and female offspring. Microarray analysis, only performed in lungs from female offspring, revealed a number of downregulated genes. Those are related to asthma and immune responses and include CCL8, CCL11, CCL24, IL4, IL6, IL10, IL13, IL1β, TNFą, and others. In a similar second hit scenario, dams were exposed to ETS from 2 weeks prior to conception until weaning of the pups. Airway reactivity was moderately increased in exposed offspring at baseline but increased dramatically together with Th2 cytokines and IgE after repeated intratracheal *Aspergillus* (*A.*) *fumigatus* instillation as compared to non-exposed controls. BHR, but not allergic sensitization, was mediated by increased expression of M1, M2, and M3 muscarinic receptors and the phosphodiesterase-4D5 isozyme as shown in inhibitor experiments [99].

Upregulation of Th2 cytokines and molecules along the Th2 pathway was further confirmed in another set of experiments with prolonged postnatal ETS exposure followed by repeated intratracheal *A. fumigatus* challenges. In contrast, goblet cell metaplasia and the expression of mucus-related genes were downregulated. The authors propose that prenatal ETS may alter the ability of mucociliary clearance [87]. In another study, prenatal ETS exposure followed by postnatal re-exposure was associated with impaired lung function, elevated pro-inflammatory cytokines in BALF and with morphological changes of the lungs. Here, the mRNA levels of metalloproteases ADAMST9 and MMP3 were upregulated, suggesting a profibrotic milieu with predisposition for obstructive pulmonary disease [100].

In an animal model for *active* smoking, similar to the human situation, active smoking during second and third trimester of pregnancy negatively affected birth weight and lung volume in murine offspring [101]. In addition, Singh *et al*. showed, in animals prenatally exposed to active maternal smoke exposure, BHR development following postnatal exposure to a single intratracheal injection of *A. fumigatus* extract in early adulthood. Interestingly, the increased BHR was not associated with more leukocyte migration or mucus production in the lung but was causally related to lower lung cyclic adenosine monophosphate levels, modulated by increased phosphodiesterase-4 enzymatic activity in the lung [86]. However, increased BHR *was* related to airway inflammation or mucus production in a different (ETS) model for maternal smoke exposure, investigated by the same research group [87].

In children, Haley *et al.* [102] investigated the effect of intrauterine smoke exposure on the expression of runt-related transcription factors (RUNX)1-3 which have critical roles in immune system development and function. In addition, genetic variations in RUNX1 were associated with BHR in asthmatic children and this association was hypothesized to be modified by intrauterine smoke exposure. Indeed, 17 out of 100 RUNX1 single-nucleotide polymorphisms (SNPs) were significantly associated with methacholine responsiveness, and the association with one of the SNPs was significantly modified by a history of intrauterine smoke exposure. Quantitative PCR analysis of immature human lung tissue suggested an increased expression of RUNX at the pseudoglandular stage of lung development after intrauterine smoke exposure. The intrauterine smoke effect on RUNX expression was further investigated in a mouse model. In this model, intrauterine smoke exposure additionally altered RUNX expression in lung tissue samples at postnatal days (P)3 and P5, in the alveolar stage of lung development. In an additional mouse study from this group, abnormal alveolarization, induced by intrauterine smoke exposure, was further associated with altered retinoic acid pathway element expression in the offspring [103]. Disrupted RUNX expression and retinoic acid signaling could therefore partly explain the consistent identification of maternal smoking as a risk factor for pediatric asthma.

Data from our own lab also indicate an effect of maternal smoking on gene transcription and lung development. Blacquiére *et al.* demonstrated that active smoking from 3 weeks prior to conception until birth resulted in lower expression of encoding forkhead box a2 (FOXA2), frizzled receptor 7 (FZD-7), epidermal growth factor (EGF), β-catenin (CTNNB1), fibronectin (FN1), and platelet-derived growth factor receptor alpha (PDGFRą) in neonatal offspring [90]. These genes are members of, or related to, the Wnt/β-catenin pathway, which plays an important role in lung branching morphogenesis [104]. In addition, in *adult* non-smoking F1 progeny from these smoking mothers, increased deposition of collagen III and smooth muscle layer thickening around the airways was found [78]. These characteristics of lung remodeling are typical for obstructive lung diseases such as asthma and COPD. The observed lung remodeling was associated with an increase in methacholine responsiveness, which is a risk factor for accelerated lung function decline in the general population and development of COPD. Since these striking differences were observed in *adult* mice that were not exposed to cigarette smoke after birth, it suggests that persistent smoke-induced epigenetic changes occurred in embryonic lungs during pregnancy.

Numerous studies investigated the effect of *maternal sensitization and/or asthma phenotype* in the offspring [96,105-107]. In some instances, combinations of allergen and pollutants were used [108]. Fedulov *et al.* demonstrated in 2011 that adoptive transfer of dendritic cells (DCs) from allergen-naive neonates from asthmatic mothers into 3-day-old mice from non-asthmatic mothers conferred increased allergen responsiveness resulting in heightened BHR and allergic inflammation. While the phenotype of ‘asthma-susceptible’ DCs was largely unaltered, DCs showed enhanced allergen-presentation activity *in vitr*o and a global increase in DNA methylation. The ‘asthma-transferring’ ability seemed to be restricted to DCs, as other investigated immune cell types did not show this ability [93]. In a model of maternal peanut food allergy, offspring exhibited higher peanut-specific IgE and histamine levels with more severe anaphylaxis after suboptimal oral peanut challenge compared to prenatally non-exposed offspring [92]. Pyrosequencing revealed hypomethylated IL-4 CpG sites in splenocytes and DNA methylation levels correlated inversely with IgE levels.

#### Experimental transgenerational epigenetics

Evidence for *trans*generational transmission of asthma risk beyond the F1 generation was demonstrated by Hollingsworth *et al.* who were the first to describe the effect of dietary methyl donors on the risk for allergic airway disease via epigenetic mechanisms [109]. In this work, methyl supplementation of pregnant and weaning dams increased the severity of allergic airway disease in the offspring but not in the mothers. There was a less prominent effect on airway eosinophilic inflammation and IgE-level in the F2 generation, and this effect was transmitted paternally. The modified risk was associated with altered DNA methylation of several genes including RUNX3, in phenotypic extremes of the F1 progeny. As mentioned earlier, RUNX3 is known to regulate T cell development and to downregulate airway eosinophilia. Nonetheless, work in mice indicates that the risk of allergic airway disease can a) be modified through epigenetic mechanisms and b) during vulnerable developmental periods only. Preconceptional exposure to intranasal *A. fumigates*, in early *vs* late pregnancy, resulted in lower IgE in grand-offspring who were re-exposed in young adulthood [83]. BALF eosinophils were increased or decreased depending on the timing of allergen exposure during pregnancy of the grandmothers. Pyrosequencing of lung DNA showed hypomethylated IL-4 CpG sites after early *A. fumigatus* exposure, whereas IFNγ was hypomethylated independent of the timing of exposure. The results from the epigenetic studies do not accord very well with the data on airway eosinophilia. However, the work demonstrates again that maternal exposures can affect the second generation and that the effects may depend from the timing of exposure during pregnancy.

Truly *trans*generational transmission of the asthma phenotype to F3 offspring was shown in a rat model of perinatal nicotine exposure [9]. The F3 generation corresponds to the great-grandchildren and is thus the first generation to be totally unexposed to the original agent. In this model, exposure of F0 dams resulted in abnormal pulmonary function, as well as altered expression of the remodeling marker fibronectin in F3. Interestingly, this transgenerational effect was sex-specific occurring exclusively in males. In the F2 generation, global DNA methylation was increased in the testes, but decreased in the ovaries, and was not changed in the lungs. H3 acetylation was increased in the lungs and testes, and H4 acetylation decreased in the lungs while it increased in the testes and ovaries, suggesting that epigenetic information predisposing to asthma may be transmitted through the germ line in this model. However, it remains unclear whether the transgenerational effect is being carried through the male or female germ line.

#### Potential mechanisms leading to transgenerational inheritance

##### Reprogramming of the epigenome

A major barrier to transgenerational inheritance is developmental reprogramming. During this process, DNA methylation, histone variants and their modifications, as well as small RNAs are all reset. This is required to remove epigenetic signatures acquired during development or imposed by the environment. It enables the zygote to acquire the totipotent state needed for the differentiation into all cell types. In mice, there are at least two rounds of genome-wide DNA methylation reprogramming. The first occurs just after fertilization, in the zygote and early cleavage stages, to erase gametic (sperm and oocyte) epigenomic marks. The next major reprogramming process occurs in the cells that will form the germ line of the developing embryo to achieve an epigenetic state distinct from somatic cells. In each reprogramming window, a specific set of mechanisms regulates erasure and re-establishment of DNA methylation [13,14]. Still, there is strong evidence for persisting transmission of the DNA methylation through the gametes to the next generation at a small number of loci in the mouse [110]. In a study investigating DNA methylation during early development of the mouse embryonic lineage *in vivo*, analysis of around 1,000 CpG islands (CGIs) within ovulated eggs showed that 15% are methylated [111]. The level of methylation was higher (25%) in sperm but the proportion of individual CpG sites methylated in CGIs in sperm was lower. By the blastocyst stage many of these methylated CGIs show some loss of methylation but not to the very low levels predicted by the accepted model for epigenetic reprogramming [112]. These were non-imprinted, non-repetitive genes (retrotransposons).

##### Parental imprinting

Parental imprinting, also known as genomic imprinting, is the process by which genes are expressed on only one of the two parental inherited chromosomes (either from the mother or father). During gamete formation, following germ line reprogramming where the paternal and maternal somatic programs are erased, parent-specific imprints are laid down in the germ line by epigenetic mechanisms [11,113]. Imprinting is displayed in only a few hundred genes in the human genome, of which most of them are located in clusters that are regulated through the use of insulators or long non-coding RNAs. However, as the imprint or memory lasts one generation, parental imprinting is not considered to be an example of transgenerational inheritance [10].

##### Chromatin proteins and epigenetic inheritance

During mammalian spermatogenesis, chromatin in differentiating germ cells is extensively remodeled, with the majority of nucleosomes being removed and ultimately exchanged by highly basic proteins named protamines. The remaining nucleosomes, unlike protamines that are exclusively re-replaced by maternal nucleosomes in the zygotes, may potentially direct certain processes of development and are thus a potential source for epigenetic inheritance through the paternal germ line [114]. Therefore, the genomic loci associated with retained nucleosomes in sperm are of great interest and have been investigated by several groups [115-118]. This has recently led to some debate on the genome-wide localization of these nucleosomes and their modification and/or variant states [119]. Two independent studies provided evidence that in mammalian sperm, nucleosomes are retained predominantly within distal gene poor regions and are depleted significantly in promoters of genes for developmental regulators [117,118]. However, these observations contradict a previous report that the retained nucleosomes in human sperm are significantly enriched at loci of developmental importance, including imprinted gene clusters, microRNA clusters, and HOX gene clusters [116]. More precise knowledge of the genome-wide distribution of the nucleosomes retained in mammalian spermatozoa is important to clarify their functional significance.

##### MicroRNAs and epigenetic inheritance

Non-protein coding RNAs (ncRNAs) are RNA sequences that regulate transcriptional and/or translational processes. Of the species of ncRNAs, the four best-characterized forms are microRNAs (miRNAs), small interfering RNAs (siRNAs), Piwi-interacting RNAs (piRNAs), and long non-coding RNAs (long ncRNAs) [120]. miRNAs are a distinct class of ncRNAs and differ from other species of ncRNAs in both how they are formed as well as their particular mechanism of action. miRNAs are processed from precursor transcripts which fold back on themselves, forming hairpin structures [121]. In general, miRNAs bind to mRNAs of protein-coding genes and direct post-transcriptional repression. Expression of miRNAs is controlled by DNA methylation of promoter-associated CpG sites of miRNA genes, as well as post-translational histone modifications [122].

Human spermatozoa are known to contain a large range of RNA molecules, including over 100 miRNAs [5,120,123,124]. Interestingly, in the spermatozoa of smokers, a total of 28 known human miRNAs were significantly differentially expressed compared with non-smokers. Ten out of the twenty-eight miRNAs had validated targets. These altered miRNAs predominantly mediated pathways vital for healthy sperm and normal embryo development, particularly cell death and apoptosis. Of interest is that in addition, 25 components of the epigenetic machinery (different modulators of DNA methylation and histone modification, such as DNMT3A, DNMT3B, and several HDACs) were shown to be targets of the altered miRNAs [5]. This means that these miRNAs could act as potential epi-miRNAs by mediating changes in DNA methylation and/or histone modification. In this way, miRNAs could affect phenotypes in future progeny.

Also in oocytes (mouse), different classes of ncRNAs have been described [125].

## Conclusions

Although a number of human studies have reported an association of prenatal smoke exposures with epigenetic changes in relation to asthma and COPD, there is very few human data available regarding the effect of grandparental exposures on disease risk in grandchildren and even less with information on epigenetic events. In addition, all available epidemiological studies address intergenerational rather than true transgenerational propagation of risk for respiratory disease. Although the few data from experimental animal models show evidence for transgenerational inheritance, also for early nutritional environmental exposures, further investigations in these models are clearly needed to unravel the underlying mechanisms. Moreover, large, well-characterized cohort studies would be needed to explore whether transgenerational inheritance indeed also occurs in humans. It will be important to investigate epigenetic signatures over time in birth cohorts and, where possible, across generations and to validate them in independent cohorts. Other important knowledge gaps that need to be addressed in the future are the need to understand the functional consequences of differentially methylated genes: even though some studies can associate subtle changes with phenotype, these may still represent an epiphenomenon. On the other hand, small changes could be important when multiple genes along a given pathway are affected. Also, epigenetic regulation is likely to differ in men and women, but this gender-specific difference has received little attention so far. In this respect, animal models can facilitate inter- and transgenerational research and may allow preclinical testing of interventions preventing deviation of epigenetic signatures to interrupt propagation of disease risks.

## Abbreviations

A: *Aspergillus*
ACSL3: acyl-CoA synthetase long-chain family member 3
ADAM33: A disintegrin and metalloprotease 33
ADRB2: adrenergic-receptor beta-2
AHRR: aryl hydrocarbon receptor repressor
Al(OH)_3_: aluminum hydroxide
ALOX12: arachidonate 12-lipoxygenase
BALF: bronchoalveolar lavage fluid
BHR: bronchial hyperresponsiveness
BLG: beta-lactoglobulin
BPA: bisphenol A
COPD: chronic obstructive pulmonary disease
CYP1A1: cytochrome P450 aryl-hydrocarbon-hydroxylase
DEP: diesel exhaust particles
DNCB: dinitrochlorobenzene
EWAS: epigenome-wide association study
FA: formaldehyde
FBZ: fenbendazol
GST: glutathione *S*-transferase
HDM: house dust mite extract
IFN: interferon
IGF2: insulin-like growth factor 2
IL-4: interleukin 4
miRNA: microRNA
MoBa: Norwegian Mother and Child Cohort Study
MS: mainstream smoke
ncRNA: non-protein coding RNA
NPSR1: neuropeptide S receptor 1
OVA: ovalbumin
PAX-5a: box 5 protein transcription factor
PGD2: prostaglandin D2
piRNA: Piwi-interacting RNA
PT: pertussis toxin
ROFA: residual oil fly ash
RUNX: runt-related transcription factors
siRNA: small interfering RNAs
SNP: single-nucleotide polymorphisms
SS: sidestream smoke
TDI: toluol-2,4-diisocyanat
Th2: type 2 helper T cell
TiO_2_: titanium dioxide
Treg: regulatory T cell
UPM: urban particulate matter
